# HUWE1-dependent DNA-PKcs neddylation modulates its autophosphorylation in DNA damage response

**DOI:** 10.1038/s41419-020-2611-0

**Published:** 2020-05-26

**Authors:** Zongpei Guo, Shaozheng Wang, Ying Xie, Yang Han, Sai Hu, Hua Guan, Dafei Xie, Chenjun Bai, Xiaodan Liu, Yongqing Gu, Ping-Kun Zhou, Teng Ma

**Affiliations:** 1 Department of Radiation Toxicology and Oncology, Beijing Key Laboratory for Radiobiology, Beijing Institute of Radiation Medicine, Beijing, 100850 P. R. China; 2 Institute for Environmental Medicine and Radiation Hygiene, School of Public Health, University of South China, Hengyang, Hunan Province 421001 P. R. China; 3 Department of Cellular and Molecular Biology, Beijing Chest Hospital, Capital Medical University/Beijing Tuberculosis and Thoracic Tumor Research Institute, Beijing, 101149 P. R. China

**Keywords:** Ubiquitins, Neddylation

## Abstract

DNA-dependent protein kinase catalytic subunit (DNA-PKcs) is the core component of DNA-PK complex in the non-homologous end-joining (NHEJ) repair of DNA double-strand breaks, and its activity is strictly controlled by DNA-PKcs phosphorylation. The ubiquitin-like protein, NEDD8 is involved in regulation of DNA damage response, but it remains mysterious whether and how NEDD8-related neddylation affects DNA-PKcs and the NHEJ process. Here, we show that DNA-PKcs is poly-neddylated at its kinase domain. The neddylation E2-conjugating enzyme UBE2M and E3 ligase HUWE1 (HECT, UBA, and WWE domain containing E3 ubiquitin protein ligase 1) are responsible for the DNA-PKcs neddylation. Moreover, inhibition of HUWE1-dependent DNA-PKcs neddylation impairs DNA-PKcs autophosphorylation at Ser2056. Finally, depletion of HUWE1-dependent DNA-PKcs neddylation reduces the efficiency of NHEJ. These studies provide insights how neddylation modulates the activity of NHEJ core complex.

## Introduction

DNA double-strand breaks (DSBs) are the most-toxic DNA lesions for all organisms. To maintain the genome stability, eukaryotic cells employ two main pathways to repair DSBs: non-homologous end-joining (NHEJ) and homologous recombination (HR)^1–3^. NHEJ functions throughout the whole cell cycle and repairs most DNA damage because it does not need the template of the homologous DNA, however, HR can only function in S/G2 cell cycle phases^4^. Upon DSBs, Ku70/80 heterodimer rapidly recognizes the broken DNA ends and recruits DNA-PKcs to form the active Ser/Thr kinase DNA-PK holo-enzyme^3–5^. As a member of PIKK (phosphatidylinositol 3-kinase-related kinases) family, DNA-PKcs targets many substrates including H2Ax, 53BP1, Ku, Artemis, itself, and so on^6,7^. When DSB occurs, DNA-PKcs is phosphorylated at the Thr2609 flanked by ABCDE cluster and Ser2056, which is surrounded by PQR cluster to initiate NHEJ pathway, and then released from broken DNA ends to make the damage sites for the subsequent repair proteins. The Thr2609 was reported to be primarily phosphorylated by ATM, which promotes end-processing, but Ser2056 is a strict autophosphorylation site to promote end-joining mediated by Ligase4, XRCC4, and XLF complex^1,4,8^. To sum up, DNA-PKcs plays an important role during the NHEJ repair pathway^9,10^.

In addition to the recruitment of DNA repair proteins, the subsequent post-translational modifications (PTM) are also essential for accurate and efficient DNA damage detection and repair^11–13^. Ubiquitin and the ubiquitin-like proteins (UBL) including SUMO (small ubiquitin-like modifier) have established roles in coordinating and regulating the cellular response to DNA DSBs^11,14,15^. A role for other UBL, especially NEDD8 (neural precursor cell expressed, developmentally downregulated 8), which is ~60% identical and ~80% homologous to ubiquitin, the highest among ub-like proteins had been detected at DNA-damaged sites induced by micro-irradiation and played an important role during DNA damage signaling^13,16,17^. Subsequent studies showed that the neddylation inhibitor MLN4924 could block DNA damage-induced ubiquitylation and removal of Ku by inactivating the SCF^FBXL12^ E3 ubiquitin ligase activity^11,18,19^. Another group showed that neddylation inhibits HR by affecting the length of CtIP-resected DNA^20^. Neddylation substrates are mainly cullins, which are components of SCF ub E3 ligases^21^. Several non-cullin substrates were identified and well characterized, however, it is unclear whether the DNA-PKcs/Ku70/Ku80 core complex could be a direct neddylation substrate and affected by neddylation.

Here, we verified NEDD8 was conjugated to the kinase domain of DNA-PKcs by HUWE1. And inhibition of DNA-PKcs neddylation could impair DNA-PKcs autophosphorylation at Ser2056, and subsequent end-joining activity. In addition, HUWE1 deletion inhibited the efficiency of NHEJ. Therefore, DNA-PKcs neddylation mediated by HUWE1 has important function of preferentially activating NHEJ pathway of DNA DSBs repair.

## Materials and methods

### Cell culture and treatment

All cell lines were originally obtained from ATCC cell bank. Hela, A549, and HEK293T were all cultured in Dulbecco's Modified Eagle’s medium supplemented with 10% fetal bovine serum (Hyclone and PAN), and maintained in a humidified incubator with 5% CO_2_ at 37 °C. Hela-shHUWE1 and Hela-shNC were generated from Hela cells, via transfecting lentivirus with shRNA-HUWE1-U6/puromycin and shRNA-NC vectors. For IR treatment, cells were irradiated with the indicated doses using ^60^Co γ-rays. Cells were then recovered in normal culture condition for the indicated time. For MLN4924 treatment, drug was administrated for 1 h at 3 μm concentration. For VP-16 treatment, cells were pretreated with VP-16 at 30 μg/ml for 1 h and then cells were harvested at indicated time.

### Antibody, plasmids, and siRNAs

All antibodies were purchased from the following sources: NEDD8 (Abcam ab81264), DNA-PKcs (Thermo Fisher Scientific MA5-13238; Abcam ab32566; Proteintech 19983-1-AP), p-DNA-PKcs S2056(Abcam ab18192), Ku70 (Santa cruz sc-17789), Ku80(Santa cruz sc-9034), UBA3(Santa cruz sc-377352), UBE2M(Abcam ab109507), UBE2F(Abcam ab185234), Flag(Sigma F3156), γH2Ax(Millipore 05-636), HUWE1(Bethyl A300-486A-T). SFB-NEDD8, SFB-NEDD8ΔG, SFB-ub, HA-NEDD8, HA-NEDD8 K48R/K60R/NoK constructs were described previously (SFB-tag: S-protein tag, FLAG epitope tag and streptavidin-binding peptide tag)^13^. His-myc-tagged-NEDD8/NEDD8ΔG and NEDP1/NEDP1 C163S constructs were generated by PCR and cloned into pcDNA3.1-Myc-HisB. Flag-tagged-DNA-PKcs-F, M and FATC fragment were cloned into pcDNA3.1. Flag-tagged HUWE1 as previously described^22^ was gifted from professor Genze Shao (Peking University School of Basic Medical Sciences). His-Myc-tagged-DNA-PKcs-M fragment K to R mutants (1–8) were generated by PCR mutagenesis and then cloned into pcDNA3.1-Myc-HisB. The siRNAs were synthesized by GenePharma (Shanghai China) and used at 10 μm final concentration. siRNA as follows: UBA3(AGAGAGAGAUUAUGAGCAA), UBE2M-1 (CAGAGGUCCUGCAGAACAA), UBE2M-2 (GAUGAGGGCUUCUACAAGA), UBE2F-1 (GGAAUAAAGUGGAUGACUA), UBE2F-2 (CAACAUAAAUACAGCAAGA), HUWE1-1 (CAUUGGAAAGUGCGAGUUA), HUWE1-2 (CUGUGAGAGUGAUCGGGAA).

### Immunoprecipitation, pull-down, and western blot

Cells pretreated as indicated were lysed with M-per mammalian protein extraction reagent (Thermo Fisher) on ice for 10 min. Then lysate was centrifuged at 12,000 × *g* for 10 min at 4 °C and the supernatant was transferred to a new 1.5 ml Eppendorf tube. SDS loading buffer was added and samples were boiled for 10 min. Then equal amounts of protein were loaded into SDS-PAGE, then separated and transferred onto nitrocellulose membrane (Millipore). 5% milk in TBST (20 mm Tris-HCl, 500 mm NaCl pH 7.5, 0.1% (v/v) Tween-20) was used for 1 h at room temperature, then the membrane was incubated with indicated antibodies overnight and washed with TBST subsequently. Bands were visualized by Imagequant LAS500 (GE).

For immunoprecipitation, cells were lysed with NETN-300 buffer (20 mm Tris-HCl Ph8.0, 300 mm NaCl, 1 mm EDTA, 0.5% Nonidet P-40) containing protease inhibitor cocktail (Roche) and 1,10-phenanthrolineon (deneddylation inhibitor, Sigma) on ice for 10 min, then supplemented with double volume of NETN-100 buffer (20 mm Tris-HCl pH8.0, 100 mm NaCl, 1 mm EDTA, 0.5% Nonidet P-40). The lysates were centrifuged at 12,000 × *g* for 10 min at 4 °C. The supernatants were collected and incubated with 1.5 μg indicated antibodies overnight at 4 °C, then protein A/G agarose (Santa Cruz) was added and incubated for 3 h at 4 °C. Then the agarose was collected by centrifuging at 1000 × *g* for 3 min and washed three times by NETN-100 buffer, and the immunoprecipitated proteins were detected by western blot.

For pull-down assay, cells transfected with indicated plasmids were lysed the same way as described above. And then the supernatants were added with S-protein agarose beads (Millipore) or Flag M2 affinity beads (Sigma) or Ni-NTA His-binding Resin (Millipore) and incubated at 4 °C for 3 h. Then the beads were collected and washed three times with NETN-100 buffer, then detected by western blot.

### In vitro neddylation assay

1 μg purified DNA-PK (from Thermo Fisher) was neddylated in the reaction buffer (50 mm Tris-HCl pH 7.5, 1 mm DTT, 2 mm NaF, 10 mm MgCl_2_, 5 mm ATP) with 1 μg of 6×His-NEDD8, 50 ng NEDD8 E1 and 200 ng NEDD8 E2 (all from Boston Biochem). The reactions were started by adding NEDD8 and samples were incubated at 30 °C for 30 min. Neddylation assays were stopped by 3 × SDS loading buffer and analyzed by western blot with anti-DNA-PKcs and anti-Ku80 antibody.

### Immunofluorescence staining assay

Cells were plated and cultured on glass coverslips before treated as indicated. After washing with phosphate-buffered saline, cells were fixed in 4% paraformaldehyde for 15 min and permeabilized in 0.25% Triton X-100 solution for 30 min at room temperature (To observe NEDD8 foci, a pretreatment step with 0.25% Triton X-100 for 10 minutes before fixation was performed). Then cells were blocked with 1% BSA and incubated with indicated primary antibodies at 4 °C overnight. Subsequently, the samples were washed and incubated with secondary antibody for 60 min. DAPI staining was performed to visualize nuclear DNA. Coverslips were mounted onto glass slides and visualized using a Nikon ECLIPSE E800 fluorescence microscope.

### Stable cell line establishment

To get HUWE1 defective Hela cells, we transfected shRNA lentiviral constructs within LV2 (U6/Puro) (GenePharma, Suzhou, Chain) and transfected cells were then selected in puromycin (2 μg/ml) for 2 weeks. The shRNA sequences were as follows: shNC: 5′-TTCTCCGAACGTGTCACGT-3′; shHUWE1-1: 5′-CATTGGAAAGTGCGAGTTA-3′; shHUWE1-2: 5′-CTGTGAGAGTGATCGGGAA-3′.

### DSB repair assay

For NHEJ-mediated repair, siNC- and siHUWE1-transfected cells were seeded on six-well plate and then transfected with linearized NHEJ report vectors. After 24 h, cells were harvested and analyzed by fluorescence activated cell sorting (FACS).

### Cell cycle analysis

shNC and shHUWE1 Hela cells were seeded on six-well plate. Next day, cells were treated with IR (4 Gy) and harvested to detect cell cycle distribution at indicated time.

### Statistical analysis

Statistical analysis was performed using one-way analysis of variance (ANOVA) in SPSS v17.0 software. The results are expressed as the mean ± standard deviation and were calculated from quantitative data obtained from three replicate experiments. The significance of the differences between two groups were determined using Student’s *t* test. The *p* values ≤ 0.05 were considered significant.

## Results

### DNA-PKcs is modified by NEDD8

**Fig. 1:**
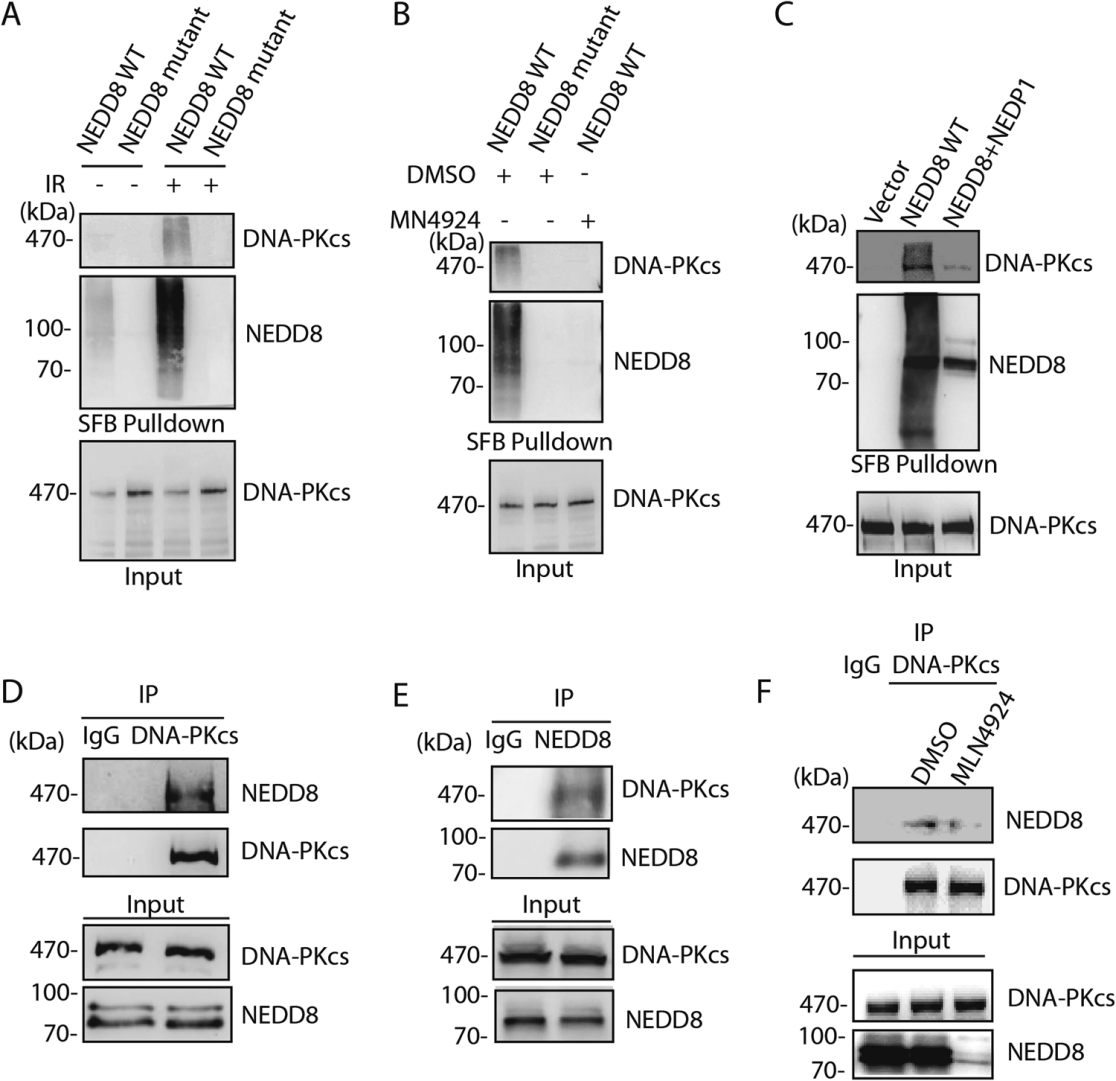
DNA-PKcs is modified by neddylation. **a** DNA-PKcs was neddylated after DNA damage. Either SFB- NEDD8 WT or SFB-NEDD8ΔG was transfected into HEK293T cells, 24 h after transfection, the cells were exposed to 10 Gy ionizing radiation. Four hours after IR treatment, cells were then harvested and subjected to Streptavidin pull-down and western blot. The blots were incubated with indicated antibodies. **b** MLN4924 treatment abolished the DNA-PKcs neddylation. SFB-NEDD8-transfected HEK293T cells were pretreated with 3 μm MLN4924 for 1 h, and cells were treated with 10 Gy IR and harvested for Streptavidin pull-down and western blot. **c** NEDP1 is required for DNA-PKcs de-neddylation. HEK293T cells were either transfected SFB-NEDD8 solely or co-transfected with NEDP1. The cells were harvested for Streptavidin pull-down and western blot 4 h after 10 Gy IR treatment. DNA-PKcs neddylation was detected by IP-western blot. **d**, **e** Endogenous DNA-PKcs was neddylated. Endogenous DNA-PKcs or endogenous NEDD8 was immunoprecipitated with specific antibodies, respectively, and the immunoprecipitated proteins were detected by either NEDD8 antibody or DNA-PKcs antibody. **f** Endogenous DNA-PKcs neddylation was inhibited by MLN4924 treatment. Hela cells were pretreated with 3 μm MLN4924 for 1 h and subjected for 10 Gy IR treatment. Six hours later the cells were harvested and processed to IP-western blotting.

Increasing evidence has shown that neddylation has important roles in DNA repair process, however, how neddylation regulates cellular DNA repair activities is not fully understood. To determine the specific role of neddylation in NHEJ pathway of DNA DSBs repair, we investigated whether DNA-PKcs/Ku70/Ku80 could be directly neddylated in cellular response to ionizing radiation. The plasmids expressing wild-type (WT) NEDD8 and NEDD8ΔG mutant with C-termini 76 Gly deletion were transfected into 239 T cells followed by streptavidin pull-down assay. The results showed that ectopic expression of wide-type NEDD8 but not the NEDD8ΔG mutant resulted in conjugation of NEDD8 on DNA-PKcs protein (Fig. 1a). The smear band possibly indicated the poly-neddylated DNA-PKcs. Alternatively, a small amount of DNA-PKcs is highly neddylated in response to DNA damage. However, neither endogenous Ku80 nor Ku70 was pulled-down by NEDD8 (Supplementary Fig. 1A). Next, when the neddylation NAE inhibitor MLN4924 was administered, it effectively abolished the NEDD8 modification on DNA-PKcs (Fig. 1b). Furthermore, when we overexpressed NEDP1 (SUMO peptidase family member, NEDD8 specific), the de-neddylation enzyme, which usually targets non-Cullin substrates, in 293 T cells, and the neddylation of DNA-PKcs was largely diminished (Fig. 1c). However, when the cells were transfected with the catalytic mutant NEDP1 C163S, the DNA-PKcs neddylation was remained (Supplementary Fig. 1B). Next, to exclude the possibility of non-specific modification owing to NEDD8 overexpression, we further confirmed the endogenous neddylation of DNA-PKcs using either DNA-PKcs or NEDD8 antibody in reciprocal immunoprecipitation. Again, as indicated, the endogenous DNA-PKcs showed modified neddylation bands (Fig. 1d, e). When the cells were treated with MLN4924, the endogenous DNA-PKcs neddylation was abolished. Besides, to gain an insight of DNA-PKcs and NEDD8 relationship, we also compared the expression profile of DNA-PKcs and NEDD8 in four independent lung cancer gene expression data sets from GEO database, and significant correlation was found as indicated (Supplementary Fig. 1C). As both DNA-PKcs and NEDD8 are important players in DNA damage response, we investigated their foci formation after IR treatment. The results showed that DNA-PKcs and NEDD8 showed ~30% colocalization in cells after DNA damage (Supplementary Fig. 1D). Collectively, these results suggest that DNA-PKcs is a novel substrate for neddylation in the processes of DNA damage response.

### DNA-PKcs is poly-neddylated mainly at its kinase domain

**Fig. 2:**
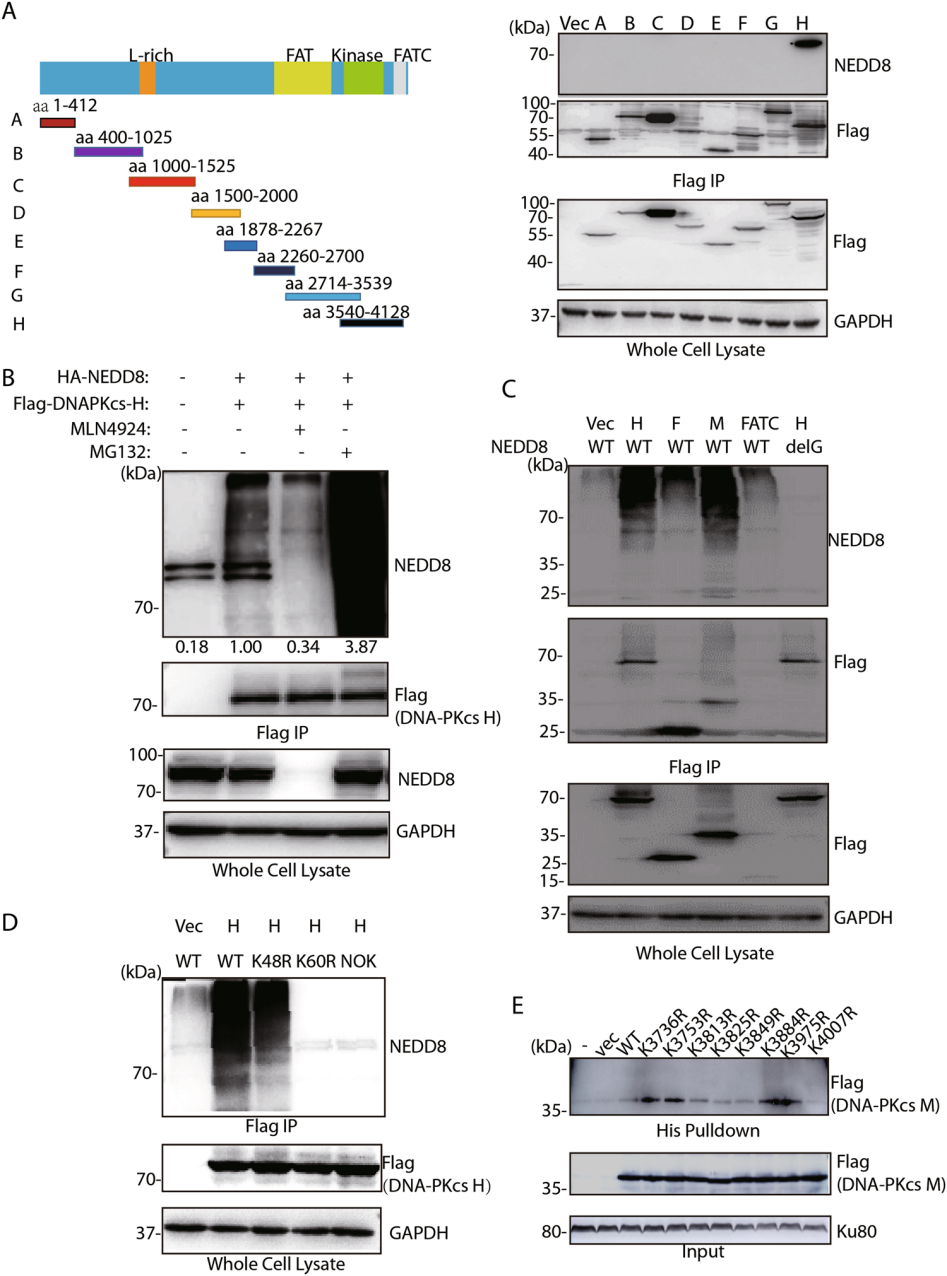
DNA-PKcs kinase domain and lysine 4007 site are modified by neddylation. **a** DNA-PKcs truncation mutant H containing kinase domain was modified by neddylation. Left panel, the schematic show of different DNA-PKcs truncation mutants. Right panel, HEK293T cells were co-transfected with different mutants and the harvested cell lysates were immunoprecipitated by Flag antibody following detection with NEDD8 antibody. **b** MLN4924 abolished the neddylation of DNA-PKcs truncation mutant H, whereas MG132 increased the DNA-PK H neddylation. DNA-PKcs mutant H was co-transfected with HA-NEDD8 and the transfected cells were treated with either MLN4924 or MG132. After treatment, cells were harvested and subjected for Flag IP and western blotting with indicated antibodies. **c** DNA-PKcs kinase domain was neddylated. Different truncation mutants of DNA-PKcs only harboring the kinase domain or FATC domain were co-transfected with HA-NEDD8 WT or HA-NEDD8ΔG plasmid. After transfection and 10 Gy IR treatment, cells were harvested and subjected for Flag IP and western blotting with indicated antibodies. **d** NEDD8 K60 mediated the poly-neddylation of DNA-PKcs. NEDD8 WT, K60R, K48R, or NOK plasmids were co-transfected with DNA-PKcs H mutant, after transfection and 10 Gy IR treatment, cells were harvested and subjected for Flag IP and western blotting with indicated antibodies. **e** K4007 is the major neddylation site of DNA-PKcs. Different K to R mutants in the DNA-PKcs kinase domain was co-transfected with His-NEDD8. After transfection and 10 Gy IR treatment, cells were harvested and subjected for Flag IP and western blotting with indicated antibodies.

The DNA-PKcs structure consists of a N-terminal region, a circular cradle unit, and a head unit with the kinase domain between FAT and FATC domains. Both the N-terminal region and the cradle unit contain HEAT repeats. Both the ABCDE and PQR phosphorylation clusters fall into the cradle unit. Next, we aimed to define which domain or site(s) was mainly neddylated in DNA-PKcs. A series of Flag-tagged-DNA-PKcs truncated mutants were constructed (Fig. 2a left panel) and transiently expressed in 293 T cells, followed by immunoprecipitation and western blotting assay. We found that only the truncation mutant H (3540aa to 4128aa) showed endogenous NEDD8 conjugation (Fig. 2a right panel). The poly-neddylated bands reduced ~70% after MLN4924 treatment while proteasome inhibitor MG132 treatment resulted in increased NEDD8 conjugation to DNA-PKcs H (Fig. 2b). To further dissect the domain within mutant H modified by neddylation, we constructed the different smaller size of functional domains based on H fragment, namely F (region between FAT domain and Kinase domain), M (kinase domain), FATC as indicated in Fig. 2c. When co-transfected with either wild-type NEDD8 or NEDD8 mutant, strong neddylation modification was only found in the kinase domain, suggesting that the neddylation site could be in kinase domain.

Like ubiquitin, NEDD8 can form chains when targeting their substrates through Lys48 and Lys60^21,23^. So, we co-transfected the cells with DNA-PK-H and NEDD8 wide-type, K48R, K60R, and NoK mutants, it was found that NEDD8 K60R mutant failed to form neddylation on DNA-PKcs-H fragment (Fig. 2d). These results suggest that DNA-PKcs is poly-neddylated at its kinase domain through Lys60 of NEDD8.

The fragment of DNA-PKcs-H (3540aa to 4128aa) contains kinase domain and FATC domain. Based on multiple sequence alignment analysis of DNA-PKcs kinase domain from different species, we found eight conservative lysine residues on this DNA-PKcs kinase domain (Supplementary Figure 2A). Neddylation on conservative lysine residues could play important roles. To examine which Lys residue(s) could be neddylated, we mutated each Lys into Arg. Each K to R mutant plasmid was co-transfected with NEDD8 into the cells, we found that the K4007R mutation attenuated kinase domain neddylation significantly (Fig. 2e). These results indicated that K4007 may be the key neddylation site on DNA-PKcs. Next, we performed mass spectrometry to confirm the neddylation of DNA-PKcs kinase domain after radiation. The K4007 site was found to be modified (Supplementary Fig. 2B).

### HUWE1 promotes DNA-PKcs neddylation

**Fig. 3:**
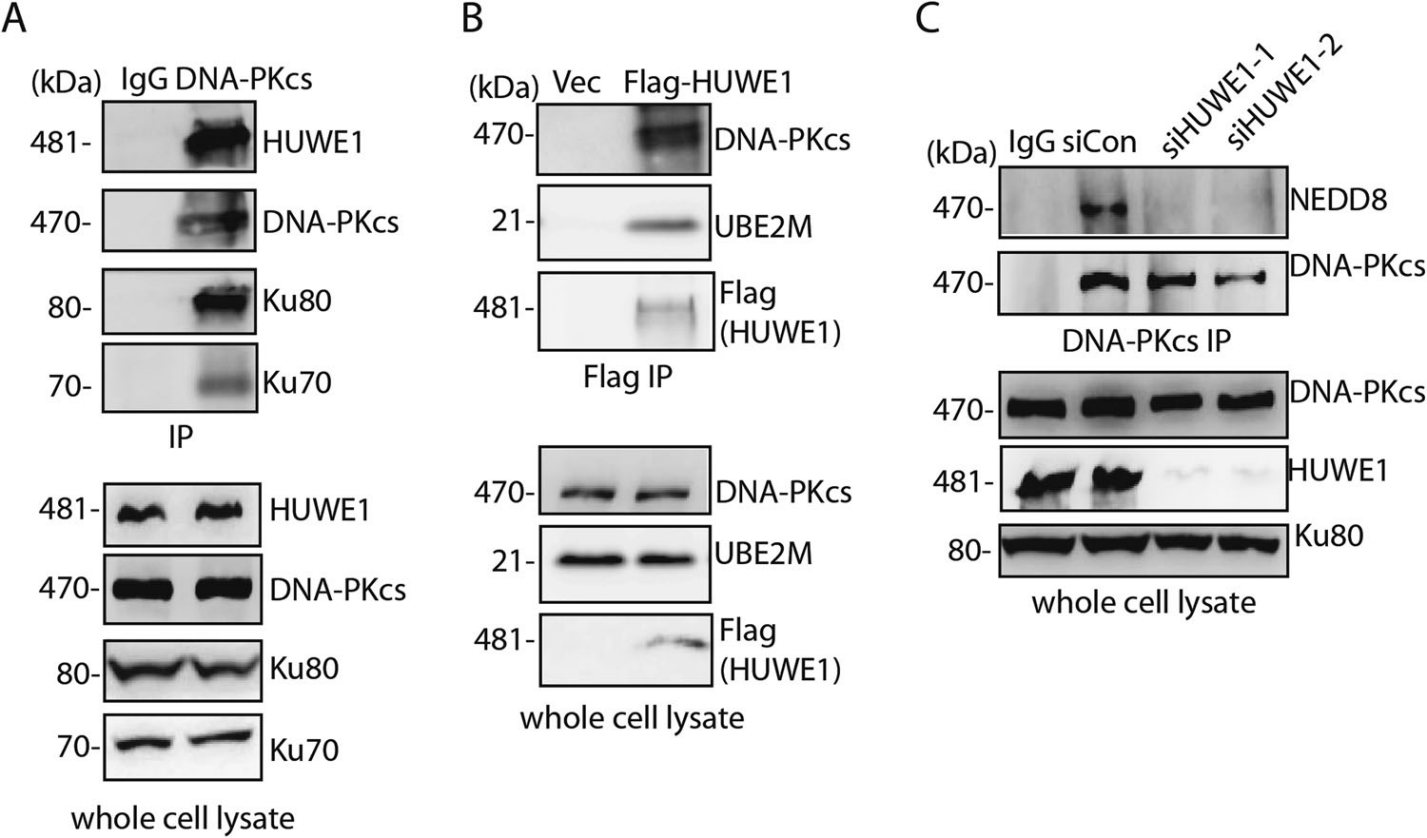
HUWE1 is the DNA-PKcs neddylation E3. **a**, **b** HUWE1 interacts with DNA-PK complex and UBE2M. Immunoprecipitation was performed with DNA-PKcs antibody or Flag-HUWE1, either HUWE1 or DNA-PKcs, Ku70/Ku80, UBE2M was detected by western blotting with indicated antibodies. **c** HUWE1 depletion abolished DNA-PKcs neddylation. HUWE1 was depleted with specific siRNAs. The depleted cells were harvested and subjected to IP with DNA-PKcs antibody following detection with NEDD8 antibody.

To date, one E1 and two E2 enzymes (UBE2M and UBE2F) have been identified that act in the neddylation cascade. And UBE2M has been demonstrated to be involved in DNA damage response and cell survival in response to ionizing radiation^13,17^. Therefore, we investigated the knockdown of neddylation E1 and E2 on DNA-PKcs neddylation, the results demonstrated that depletion of UBA3 and UBE2M resulted in decreased DNA-PKcs neddylation but not UBE2F (Supplementary Fig. 3A). The in vitro neddylation assay using purified E1/E2/NEDD8/DNA-PK also showed ~10% neddylation signal above on DNA-PKcs protein (Supplementary Fig. 3B). UBE2M together with its partner E3 determines the substrate specificity in neddylation system. To find the E3 ligase for DNA-PKcs neddylation, we performed mass spectrometry analysis of the immunoprecipitation product of Flag-DNA-PKcs kinase domain (3719aa to 4015aa) fragment (Supplementary Fig. 3C). Four potential E3 ligases including HUWE1, RNF126, TRIM21, and UBR1 were identified from the mass spectrometry. Next siRNAs against HUWE1, RNF126, TRIM21 and UBR1 were transfected into HEK293T cells, and DNA-PKcs immunoprecipitation was performed to detect the neddylation. The results showed that only in HUWE1 knockdown cells, DNA-PKcs neddylation was abolished (Supplementary Fig 3D). Next, we investigated the subcellular colocalization of DNA-PKcs and HUWE1 in IR treated cells, and the results showed that DNA-PKcs and HUWE1 shared 30–40% foci colocalization after DNA damage (Supplementary Fig. 3E). Furthermore, In Co-IP experiment, HUWE1 was found to interact with DNA-PKcs and the neddylation conjugating enzyme UBE2M (Fig. 3a, b). Ku70/Ku80 was also found in the DNA-PKcs/HUWE1 complex. Again, when HUWE1 was depleted by siRNA, DNA-PKcs neddylation was abolished (Fig. 3c). These results verified HUWE1 as the DNA-PKcs neddylation E3.

### Neddylation promotes autophosphorylation of DNA-PKcs at Ser2056

**Fig. 4:**
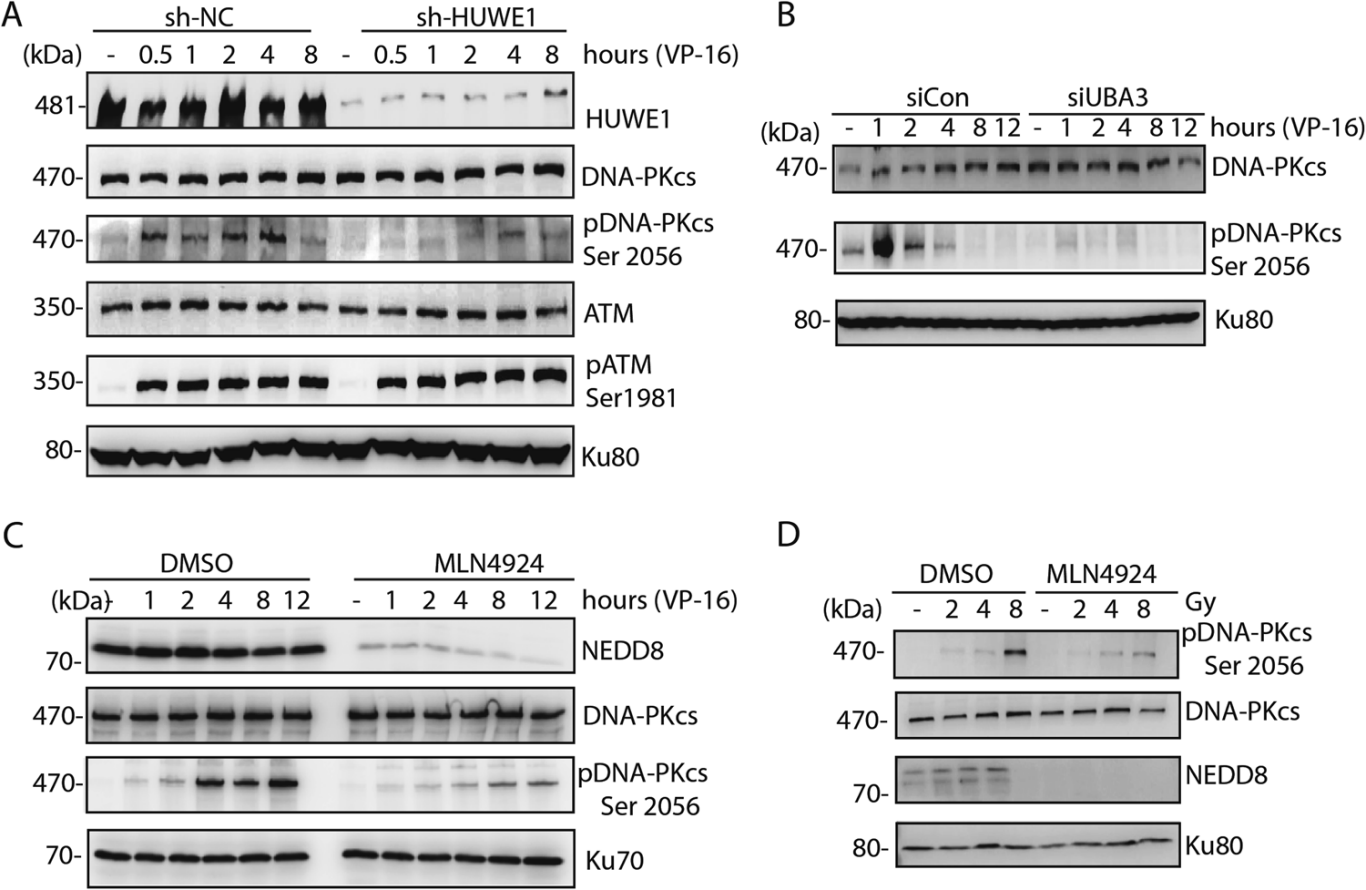
DNA-PKcs neddylation promotes DNA-PKcs Ser2056 phosphorylation. **a** HUWE1 depletion diminished DNA-PKcs Ser2056 phosphorylation. HUWE1 shRNA-depleted cells were treated with VP-16 and harvested at indicated timepoints, then DNA-PKcs Ser2056 phosphorylation or ATM Ser1981 phosphorylation was examined in western blotting. **b** UBA3 knockdown abolished DNA-PKcs Ser2056 phosphorylation. UBA3 siRNA-depleted HeLa cells were treated with VP-16 and harvested at indicated timepoints, then DNA-PKcs Ser2056 phosphorylation was examined in western blotting. **c**, **d** MLN4924 treatment diminished DNA-PKcs Ser2056 phosphorylation after VP-16 or IR treatment. HeLa cells were pretreated with 3 μm MLN4924 for 1 h and subjected to VP-16 treatment or different doses of IR treatment, then the cells were harvested followed by western blotting with DNA-PKcs Ser2056 antibody or indicated antibodies.

As DNA-PKcs autophosphorylation is the key event in DNA damage response and NHEJ, we checked the effect of neddylation inhibition on DNA-PKcs Ser2056 autophosphorylation. Knockdown of HUWE1 diminished the DNA-PKcs Ser2056 phosphorylation in VP-16-induced DNA damage response (Fig. 4a). Similar results were observed in neddylation E1 inhibition by UBA3 siRNA and MLN4924, respectively (Fig. 4b–d) in the response to either VP-16 or IR-induced DNA damage. These results indicated that DNA-PKcs neddylation is important for DNA damage-induced DNA-PKcs Ser2056 phosphorylation.

### Neddylation of DNA-PKcs regulates NHEJ

**Fig. 5:**
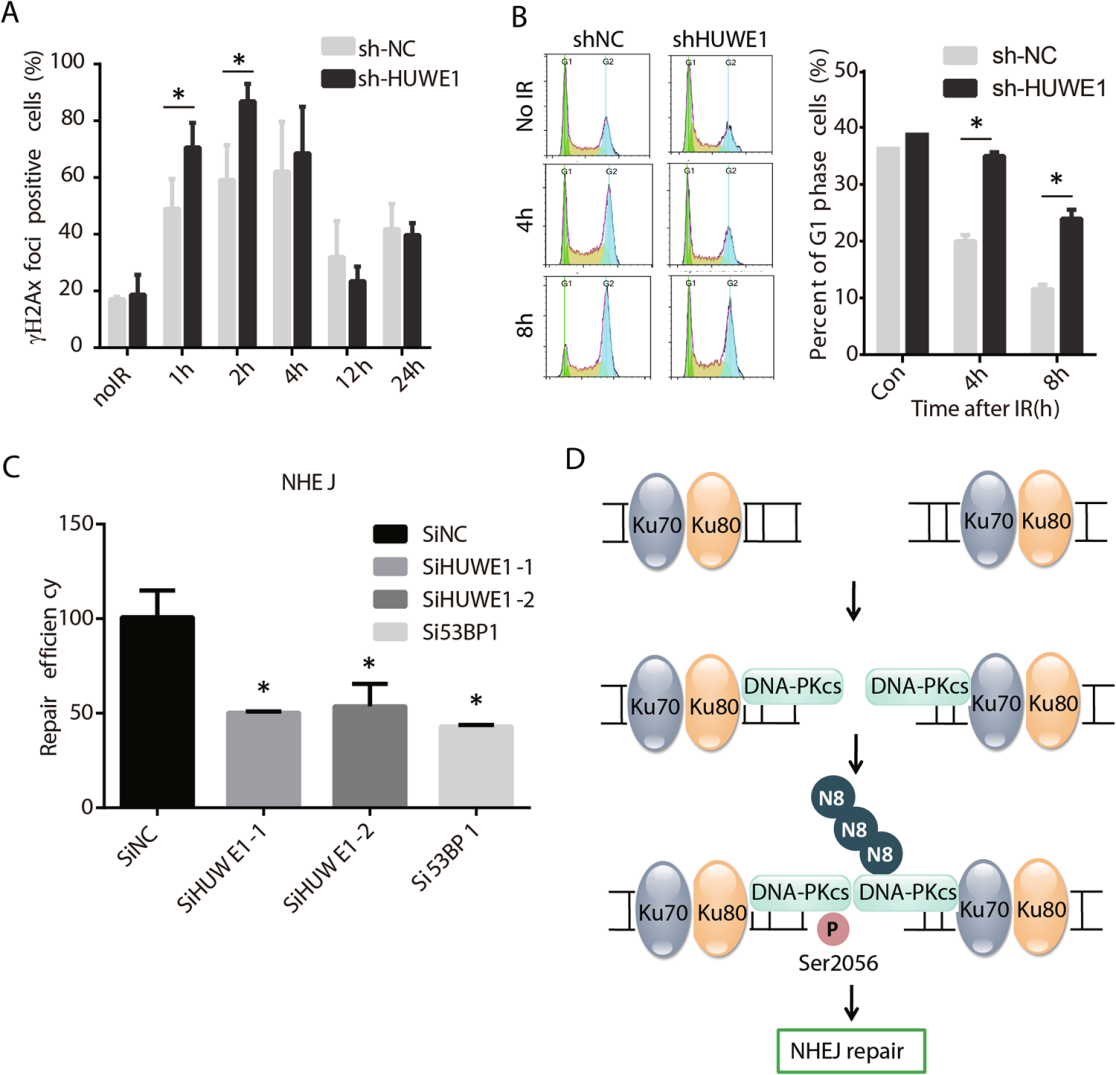
DNA-PKcs neddylation modulates NHEJ repair. **a** Increased γH2AX foci in HUWE1-depleted cells. shHUWE1 and control cells were treated with 10 Gy IR and stained with γH2AX antibody at different timepoints after IR. **b** Depletion of HUWE1-dependent DNA-PKcs neddylation resulted in cells being arrested in G1 phase. **c** Depletion of HUWE1-dependent DNA-PKcs neddylation inhibited NHEJ repair. Both two HUWE1 siRNAs showed statistical significance compared with control (*p* < 0.05). 53BP1 knockdown was used as positive control here. **d** The schematic depiction of how HUWE1-dependent DNA-PKcs neddylation affects NHEJ repair.

To determine whether DNA-PKcs neddylation affects NHEJ repair, we depleted HUWE1 by lentivirus-mediated knockdown and detected the γH2Ax foci after irradiation. The results indicated that the significant higher level of γH2Ax foci was detected in the HUWE1-depleted cells as compared to control cells at 1 h and 2 h after IR treatment (Fig. 5a), suggesting the DNA DSBs repair efficiency was possibly decreased after HUWE1 knockdown. As NHEJ functions predominantly in G1 phase, next we examined the cell cycle change in HUWE1-depleted cells. As shown in Fig. 4b, more G1 cells were arrested after 4 Gy IR treatment (Fig. 5b). Furthermore, NHEJ pathway activity was assayed in HUWE1 knockdown cells. It showed that the NHEJ repair efficiency was decreased in HUWE1 knockdown cells (Fig. 5c). These results indicated that HUWE1-mediated DNA-PKcs neddylation may play important functions in NHEJ repair pathway.

## Discussion

In the current study, we discovered the new mechanisms for DNA-PKcs activation after DNA damage. As well acknowledged, when DSBs take place, Ku70/Ku80 are first recruited to the DSB sites, and then DNA-PKcs was recruited. Here, we found that E3 ligase HUWE1 promotes the DNA-PKcs neddylation and modulates the DNA-PKcs autophosphorylation, further affects the NHEJ repair (Fig. 5d).

NEDD8 shares most homology with Ubiquitin and modifies its substrate through canonical three step cascade, namely E1-E2-E3. The main substrate for neddylation in cells are Cullin family members and later on other substrates were also found. As for DNA-PKcs and NEDD8, the association between these two proteins was first mentioned by Zhenqiang Pan et al. study through mass spectrometry analysis in NEDD8 overexpressed cells^24^. However, it is increasingly believed that overexpressed NEDD8 in cells could use ubiquitin to form chain. Thus, it is careful to claim a novel substrate of NEDD8. Here, we demonstrate the endogenous DNA-PKcs neddylation. Both the E2 and E3 were also confirmed. UBE2M is required for NEDD8 conjugating and HUWE1 functions as DNA-PKcs neddylation E3 ligase. Besides, NEDP1 was also verified to deneddylate DNA-PKcs neddylation, which is in accordance with that NEDP1 could deneddylate non-culllin substrates^25,26^. Moreover, we used different truncation mutation of DNA-PKcs and identified the kinase domain and lysine 4007 as the major neddylation sites.

Neddylation E3 ligases can catalyze NEDD8 transfer from the E2 enzyme onto the targets^21^. Among all known NEDD8 E3s, the best characterized are RBX1 and RBX2, which belong to the RBX family^27^. RBX1 catalyzes neddylation of Cullin1, Cullin2, Cullin3, and Cullin4 together with UBE2M, whereas RBX2 pairs with UBE2F for Cullin5 neddylation^27–29^. In addition to RBX, several other neddylation E3s have been identified or implicated, such as MDM2^30^, c-CBL^31^, RNF111^13^, IAPs^32^, TFB3^33^, TRIM40^34^, DCNL1-5, and FBXO11^27^ and HUWE1^35^. Here, we performed mass spectrometry using DNA-PKcs kinase domain as bait and identified HUWE1 as DNA-PKcs neddylation E3.

Now that, HUWE1 interacts with and neddylates DNA-PKcs at its kinase domain, how neddylation affects the activity of DNA-PKcs is the most intriguing question. As a member of PIKK family, DNA-PKcs is recruited to broken DNA ends by Ku70/80 heterodimer and form the DNA-PK holo-enzyme upon DNA damage. The DNA-PKcs dimer promoted by Ku and DNA facilitates trans-autophosphorylation at the DSB. Then, the activated DNA-PK holo-enzyme has an important role during NHEJ^1,9,36^. DNA-PKcs can be auto-phosphorylated at ABCDE cluster flanking Thr2609 and the PQR cluster around Ser2056 during DNA damage response. However, recent studies suggest that the Thr2609 is primarily phosphorylated by ATM or ATR under different cellular stress, whereas Ser2056 is auto-phosphorylated, but both are important for DNA repair and dissociation from damage sites. It was reported previously that phosphorylation of DNA-PKcs at Ser2056 is important for DNA repair and responsible for DNA-PKcs dissociation from damage sites^1,3,9,36^ and it is necessary for completion of NHEJ repair.

Here, we found that HUWE1-mediated DNA-PKcs neddylation affects Ser2056 autophosphorylation status. Previous study has confirmed that neddylation promotes the Ku70 ubiquitination and release from DNA damage sites^37^. Here, DNA-PKcs neddylation in kinase domain could directly affect the structure of DNA-PK for more liable to be transactivated at the Ser2056 site and then facilitate its release from DNA damage sites following Ku70/Ku80. However, more work is needed to illustrate how Ser2056 is phosphorylated from structural biology.

In summary, DNA-PKcs neddylation functions as novel DNA-PKcs PTM, and plays important roles in DNA-PKcs Ser2056 phosphorylation. HUWE1-dependent DNA-PKcs neddylation serves as new manner of mechanistic regulation of NHEJ repair activity.

## Supplementary information

Supplementary Information

Supplementary Figure 1

Supplementary Figure 2

Supplementary Figure 3
