# Supplementary material for: HUWE1-dependent DNA-PKcs neddylation modulates its autophosphorylation in DNA damage response
Source: Cell Death Dis. 2020 May 26;11(5):400. doi: 10.1038/s41419-020-2611-0 (PMC7250858; doi:10.1038/s41419-020-2611-0)
Supplement: Supplementary file 1 — Supplementary Information [file 41419_2020_2611_MOESM1_ESM.docx]

**Supplementary Materials**

**Materials and methods**

**LC–MS/MS Analysis of Nedd8-Modified DNA-PKcs protein**

A stable cell line expressing Flag-DNA-PKcs H (aa 3520-4128) was grown in 10-cm tissue culture dishes until confluency. Because of low concentration of Nedd8 conjugated proteins, purification was scaled up using 50 plates of the cells. 35mL lysates were purified by Flag antibody conjugated agarose and anti-NEDD8-coupled protein A beads.

Coomassie-stained band corresponding to neddylated DNA-PKcs H was treated with 12.5 ng/μl sequencing-grade trypsin (Promega), and peptides were extracted from the gel by repeated dehydration with acetonitrile (Fisher Scientific) and swelling with 50 mM ammonium bicarbonate (Sigma Aldrich), as previously described ([Maine et al., 2010](#_ENREF_3)). Data obtained from MS-MS are initially analyzed against the IPI database v. 3.41 and appended with decoy database using X!Tandem software with a k-score plug-in. Next, a more restricted analysis is recommended using a target protein database containing predicted peptides from tryptic digestion of the DNA-PKcs-H, NEDD8 and common contaminants. The potential modifications of amino acids that may lead to changes in mass and/or charge were analyzed in the MS-MS data. In particular, oxidation of methionine (+15.9949 Da), carbamidomethylation of cysteines (+57.0214 Da) and neddylation of lysines (diglycine, +114.0469 Da) are considered as variable modifications in the analysis. Manual verification of the MS-MS spectra corresponding to peptides with a mass shift corresponding to GG or LRGG is considered as evidence of neddylation([Jones et al., 2008](#_ENREF_2)).

**Supplementary Figure legends**

**Supplementary Figure S1. DNA-PKcs is neddylated (A)** Ku70 and Ku80 are not substrate for neddylation. Either SFB- NEDD8 WT or SFB-NEDD8ΔG was transfected in HEK293T cells, 24h after transfection, the cells were exposed to 10Gy ionizing radiation. Four hours after IR treatment, cells were then harvested and subjected to Streptavidin pulldown and western blot. The blots were incubated with indicated antibodies. **(B)** NEDP1 is required for deneddylation of DNA-PKcs. HEK293T cells were either transfected SFB-NEDD8 solely or co-transfected with SFB-NEDD8 and NEDP1 or NEDP1 C163S mutant. The cells were harvested for Streptavidin pulldown and western blot 4h after IR treatment. (C) Correlation analysis of DNA-PKcs and NEDD8 in 4 independent lung cancer gene expression databases from Gene Expression Omnibus. GSE27262 (Gene expression profiling of Non-small cell lung cancer in Taiwan); GSE10445 (MERLION LUNG CANCER STUDY); GSE43580 (SBV - Gene Expression Profiles of Lung Cancer Tumors - Adenocarcinomas and Squamous Cell Carcinomas); GSE8894 (Prediction of Recurrence-Free Survival in Postoperative NSCLC Patients—a Useful Prospective Clinical Practice) (D) DNA-PKcs colocalized with NEDD8 after DNA damage. HeLa cells were irradiated with 10 Gy of IR and stained with antibodies against NEDD8 and DNA-PKcs. Scale bar = 20 μm. The percentage of foci colocalization was summarized in the histogram. Data are represented as mean +/- SEM.

**Supplementary Figure S2. Lysine 4007 of DNA-PKcs kinase domain is major site for neddylation. (A)** Multiple sequence alignment of DNA-PKcs kinase domain in 5 mammalian species showed conservative lysines including K4007. **(B)** Raw data of mass spectrometry is presented to demonstrate the K4007 neddylation.

**Supplementary Figure S3. HUWE1 is the E3 for DNA-PKcs neddylation. (A)** Knockdown of UBE2M and UBA3 abolished DNA-PKcs neddylation. siRNAs specific to UBE2M, UBE2F and UBA3 were transfected into HeLa cells respectively, 24 hours after transfection, cells were irradiated with 10Gy and then harvested for IP with DNA-PKcs antibody. (B) In vitro neddylation assay showed around 10% modification of DNA-PKcs. (C) Coomassie brilliant blue staining of Flag-DNA-PKcs H immunoprecipitation with or without IR. (D) HUWE1 knockdown diminished DNA-PKcs neddylation. siRNAs specific to the potential E3s from mass spectrometry analysis were transfected into HeLa cells respectively, 24 hours after transfection, cells were irradiated with 10Gy and then harvested for IP with DNA-PKcs antibody. (E) DNA-PKcs colocalized with HUWE1 after DNA damage. Representative immunofluorescence micrographs of HeLa cells untreated or treated with 10Gy IR. Scale bar = 20 μm. Quantifications showing the percentage of DNA-PKcs foci co‐localizing with HUWE1 foci and, reciprocally, the percentage of HUWE1 foci co‐localizing with DNA-PKcs foci. Only cells with more than five foci were analyzed. The average of three experiments, with standard errors, is shown.
