# Supplementary figures and images for: HUWE1-dependent DNA-PKcs neddylation modulates its autophosphorylation in DNA damage response
Source: Cell Death Dis. 2020 May 26;11(5):400. doi: 10.1038/s41419-020-2611-0 (PMC7250858; doi:10.1038/s41419-020-2611-0)

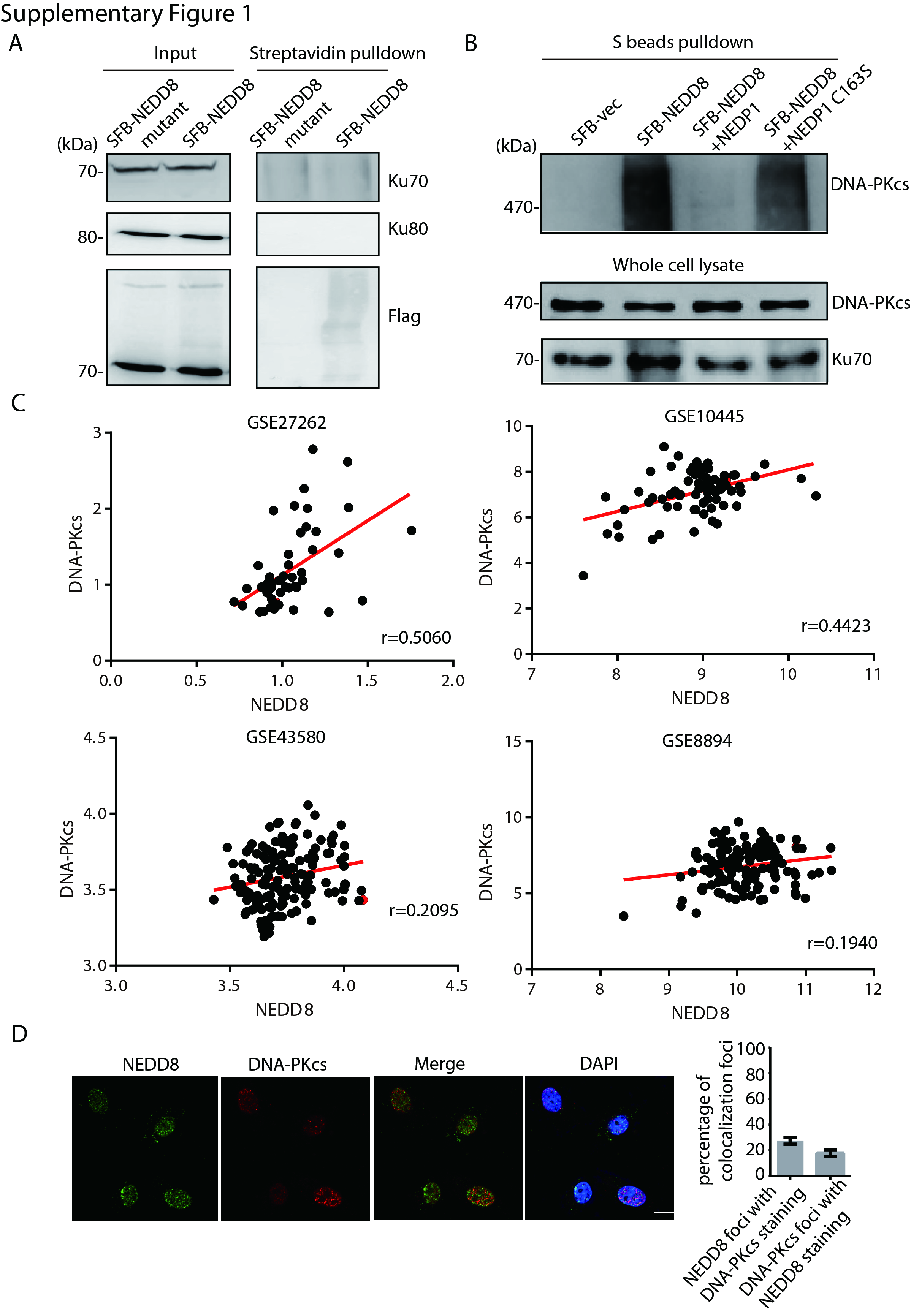

Supplement: Supplementary file 2 — Supplementary Figure 1 [file 41419_2020_2611_MOESM2_ESM.tif]

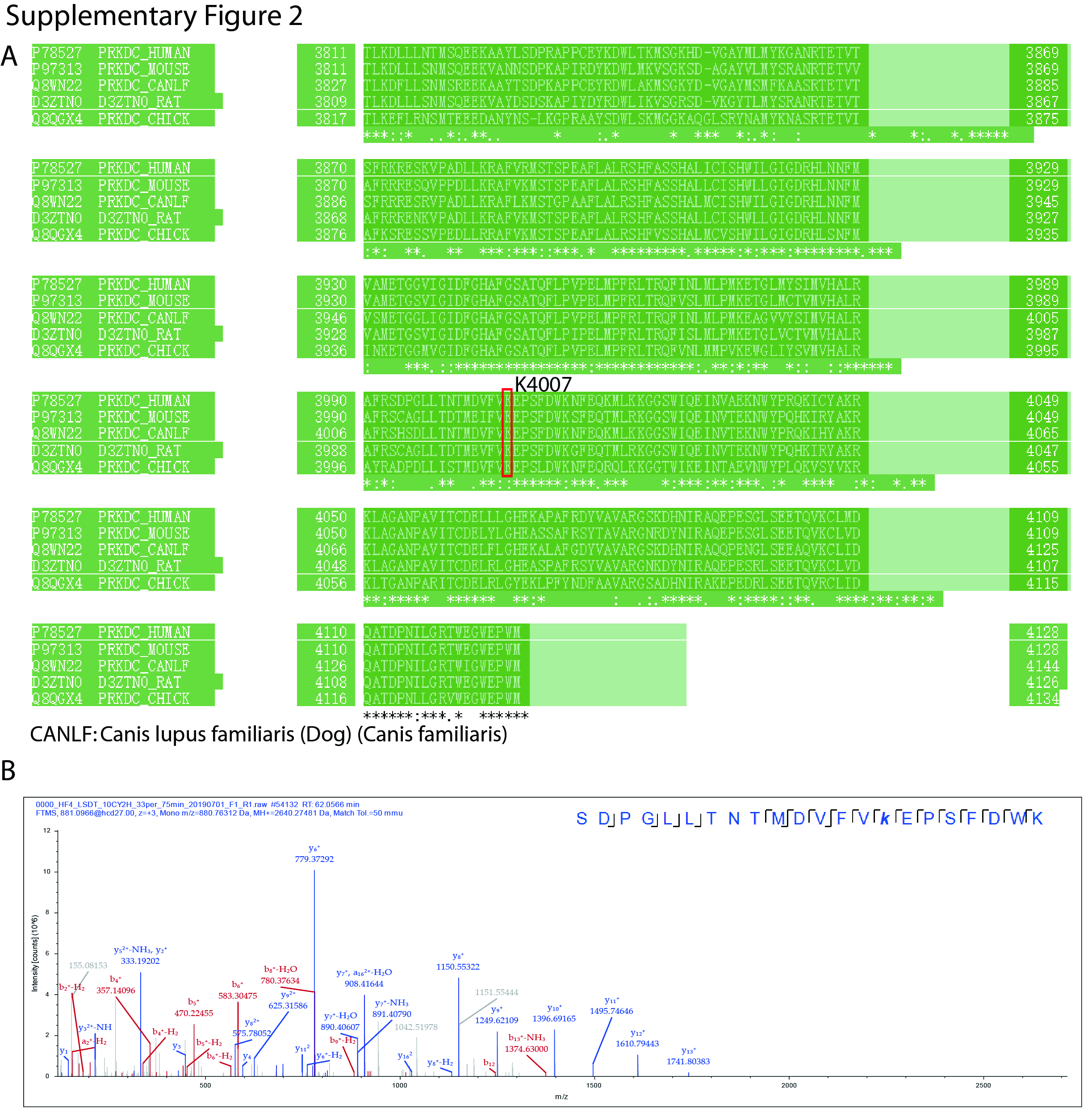

Supplement: Supplementary file 3 — Supplementary Figure 2 [file 41419_2020_2611_MOESM3_ESM.tif]

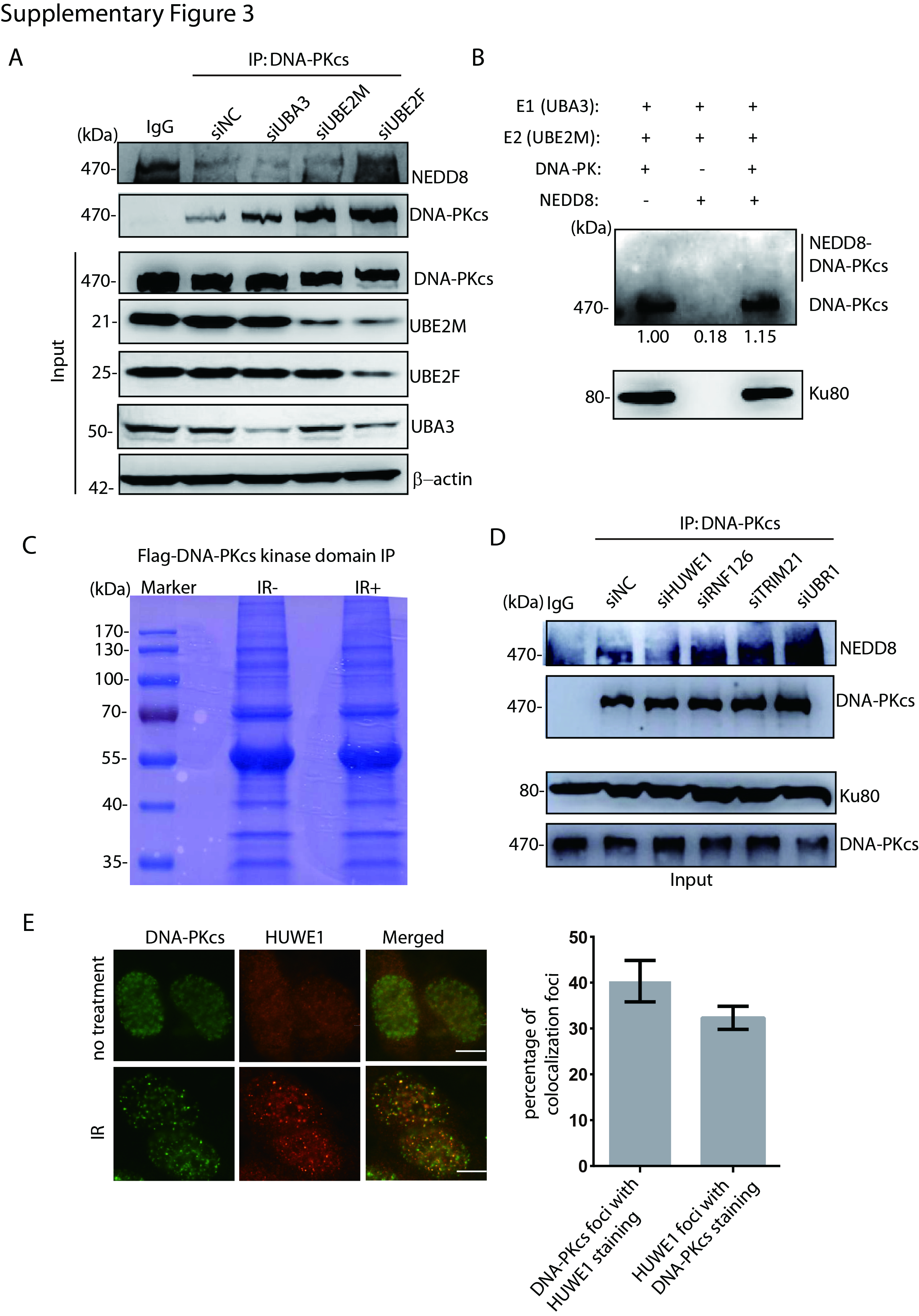

Supplement: Supplementary file 4 — Supplementary Figure 3 [file 41419_2020_2611_MOESM4_ESM.tif]
